# Utilization of mobile phones for accessing menstrual regulation services among low-income women in Bangladesh: a qualitative analysis

**DOI:** 10.1186/s12978-016-0274-1

**Published:** 2017-01-14

**Authors:** Chelsea Jordan Messinger, Ilias Mahmud, Sushama Kanan, Yamin Tauseef Jahangir, Malabika Sarker, Sabina Faiz Rashid

**Affiliations:** 1 Yale College, Yale University, New Haven, CT USA; 2 James P Grant School of Public Health, BRAC University, 5th Floor, (Level-6), icddr,b Building, 68 ShahidTajudin Ahmed Sharani, Mohakhali, Dhaka, 1212 Bangladesh; 3 Present Address: Harvard Medical School, Boston, MA 02115 USA

**Keywords:** Menstrual regulation, Abortion, mHealth, Reproductive health, Maternal health, Community health services, Family planning, Low-income population, Bangladesh

## Abstract

**Background:**

As many as one-third of all pregnancies in Bangladesh are unplanned, with nearly one-half of these pregnancies ending in either menstrual regulation (MR) or illegal clandestine abortion. Although MR is provided free of charge, or at a nominal cost, through the public sector and various non-profits organizations, many women face barriers in accessing safe, affordable MR and post-MR care. Mobile health (mHealth) services present a promising platform for increasing access to MR among low-income women at risk for clandestine abortion. We sought to investigate the knowledge, attitudes and practices regarding mHealth of both MR clients and formal and informal sexual and reproductive healthcare providers in urban and rural low-income settlements in Bangladesh.

**Methods:**

A total of 58 interviews were conducted with MR clients, formal MR providers, and informal MR providers in four low-income settlements in the Dhaka and Sylhet districts of Bangladesh. Interview data was coded and qualitatively analysed for themes using standard qualitative research practices.

**Results:**

Our findings suggest that low-income MR clients in Bangladesh have an inadequate understanding of how to use their mobile phones to obtain health service information or counselling related to MR, and correspondingly low levels of formal or informal mHealth service utilization. Few were aware of any formal mHealth services in place in their communities, despite the fact that providers stated that hotlines were available. Overall, MR clients expressed positive opinions of mHealth services as a means of improving women’s access to affordable and timely MR. Formal and informal MR providers believed that mobile phones had benefits with respect to information dissemination and making appointments, but emphasized the necessity of in-person consultations for effective sexual and reproductive healthcare.

**Conclusions:**

We report low utilization yet high acceptability of mHealth services among low-income MR clients in Bangladesh. Expanding formal and informal mHealth services targeted towards MR – and increasing publicity of these services in low-income communities – may help increase timely access to accurate MR information and formal providers among women at risk for clandestine abortion. While expanding formal and informal mHealth services for SRHR in Bangladesh may be useful in disseminating information about MR and connecting women with formal providers, in-person visits remain necessary for adequate treatment.

## Plain English summary

One-third of all pregnancies in Bangladesh are unplanned, and one-half of these pregnancies end in either legal menstrual regulation (MR) or illegal unsafe abortion. Although MR is provided for free or at nominal fees by the government and non-profit organizations, many women encounter difficulties accessing safe, affordable MR and post-MR care. Healthcare services provided through mobile phones, called mHealth, may help increase access to MR among poor women who are at risk for unsafe abortion.

We sought to understand the knowledge, attitudes and practices of MR clients living in urban and rural low-income settlements in Bangladesh and their reproductive healthcare providers regarding mHealth. Interviews were conducted in four low-income settlements in Dhaka and Sylhet districts with MR clients.

Most of the MR clients we interviewed do not use their mobile phones to get information or counselling about MR. Few were aware of formal mHealth services such as hotlines available in their communities, and most did not use their phones to contact providers for MR-related advice. Despite this, MR clients felt that mHealth services could improve timely and affordable access to MR. Formal and informal MR providers alike believed that mobile phones were useful for giving information about MR and making appointments, but emphasized the importance of in-person consultations.

Expanding formal and informal mHealth services targeted towards MR may help increase timely access to MR among women at risk for unsafe abortion in low-income communities in Bangladesh. These services must be well-publicized in order for women to use them. However, mHealth services cannot replace in-person consultations.

## Background

The maternal mortality ratio (MMR) in Bangladesh has declined markedly over the past several decades, with a 60% or greater overall reduction from 1990 to 2011 [1]. According to the 2010 Bangladesh Maternal Mortality and Health Care Survey, there were 194 maternal deaths for every 100,000 live births in Bangladesh (95% CI: 149-238) [2], down from 322 (95% CI: 259-391) in 2001 [3]. The country was expected to achieve the UN Millennium Development Goal 5A of a ¾ reduction by 2015, to 143 deaths per 100,000 live births [4].

Part of Bangladesh’s success in lowering the maternal mortality ratio can be attributed to the country’s allowance of a procedure called “menstrual regulation” (MR), which was introduced into the national family planning program in 1979 [5]. Although Bangladesh’s penal code, which dates back to 1860, states that abortion is illegal except for in cases where it is required to save the life of the mother [6], the MR provision allows formally-trained providers to “establish non-pregnancy” through manual vacuum aspiration or oral mifepristone-misoprostol regimens up to 8-10 weeks after the last menstrual period, depending on the type of provider [5].

The rate of menstrual regulation procedures conducted in Bangladesh is on the rise, according to data from the Bangladesh Demographic and Health Survey of 1999–2000. In fact, it is estimated that 5% of all married Bangladeshi women undergo MR in their lifetime [7]. Despite the fact that MR services in Bangladesh are provided free of charge or for a subsidized charge through the public sector and various non-profit organizations (NGOs), a number of studies have found that many women face barriers in accessing safe, affordable MR and post-MR care. Singh et al. in 1997 estimated that 33% of all births in Bangladesh are unplanned, and 45% of these unplanned pregnancies end in either menstrual regulation or unsafe abortions [8]. Furthermore, as many as 25% of all clients who present at MR clinics are refused services for various reasons [9].

One of the barriers that Bangladeshi women face in accessing safe MR is an information gap about how, when and where to access MR services provided by formally trained providers. Many women either do not know about MR, or have an inaccurate understanding of it. Studies have shown that only 9% of MR clients are told about the service by trained family planning workers, and that the majority of women rely on unofficial sources such as relatives, friends, and neighbours for information about MR [10]. This information gap is particularly prominent among low-income women. One study conducted in 2007 showed that one-fifth of all married Bangladeshi women had never heard of MR, and that among the poorest and least educated women this proportion was one in four [11]. Moreover, many women do not understand the difference between safe MR procedures performed by trained providers and unsafe abortions performed by informal providers, and may go to informal providers in an attempt to keep their procedures secret [12]. As a result of these barriers and of the 10-week gestational limit on MR, many women resort to seeking out induced abortions from informal providers who are untrained and unregulated. In fact, it has been reported that as many as 70–75% of Bangladeshi women seeking termination of pregnancy go to informal providers [13]. Dissemination of proper information on MR services is therefore an important tactic for reducing dependency on informal providers and increasing access to safe MR care.

### Mobile health technology

The use of mobile technology for health, termed “mHealth,” has the potential to strengthen health systems in low-and middle-income settings through improved access to knowledge and information about health services [14]. With respect to maternal and reproductive health, both formal mHealth programmes and informal uses of mobile phone technologies can connect women to care and reduce the response time in obstetric emergencies. Studies on mobile health services in Bangladesh have documented 19 separate mHealth initiatives involving the dissemination of health information from health authorities to subscribers [14]. The services offered by NGO’s vary and include, for example, the use of tele-employees who provide door-to-door services, empowering community health workers with simple mobile phones that they can use to gather client information in real time.

Sexual and Reproductive Health and Rights (SRHR) initiatives in Bangladesh, like those of the non-governmental organization BRAC, are already piloting mHealth programmes to enhance service delivery to pregnant women during routine antenatal care, labour and delivery, and for obstetric emergencies [15]. BRAC’s Manoshi programme for urban maternal, neonatal and child health, for example, empowers community health workers with simple mobile phones that they can use to gather client information in real time. The feedback provided by the Manoshi mHealth system enables health workers to obtain remote consultation in order to prioritize treatments (e.g. for high-risk pregnancies) and identify clients who need early interventional or emergency treatment for childbirth complications [15].

In the absence of formal programmes, pregnant women in rural areas of Bangladesh are also using mobile phones to obtain medical advice, arrange for transportation to emergency obstetric facilities, and ask for financial support [16]. However, little is known about whether Bangladeshi women use mobile technology to obtain information and treatment advice regarding MR services. Given the scarcity of accurate information about MR among low-income women and the stigma associated with the procedure, the use of mobile phone technology to disseminate treatment advice and information about MR, both through formal hotlines and through informal communication with healthcare workers, presents an innovative tactic for narrowing the information gap in this population. The objective of the present study was to understand the knowledge, attitudes and practices of women living in urban and rural low-income settlements in Bangladesh regarding the use of mobile phone technology for accessing MR services. We also sought to understand the perceptions of both formal and informal MR service providers regarding the utility of mobile phone technology for this purpose.

## Methods

A qualitative approach was used to gather in-depth information about the perceptions of close-to-community (CTC) MR service providers and the women accessing these services (hereafter referred to as MR clients) on the utility of mobile phone technology for accessing MR services. The qualitative data used in this analysis was collected by a larger study conducted for an international consortium project called REACHOUT (http://www.reachoutconsortium.org/). The purpose of the larger study was to understand the context in which CTC providers currently operate in urban and rural low-income settlements in Bangladesh with respect to sexual and reproductive health [17].

### Study site locations

Two urban low-income settlements from Dhaka district (containing the capital city, Dhaka, which has the largest urban low-income settlements in Bangladesh) and one urban and rural low-income settlement from Sylhet district (a largely traditional and conservative district that is low-performing on SRH indicators [7]) were selected as study sites. MR clients and key informants were recruited for in-depth interviews from two NGO clinics, both of which provide SRHR services including MR in our study sites.

### Sampling and data collection

To begin, a CTC health service provider mapping was conducted in the study sites in order to list CTC providers and identify their locations at all four study sites. Data collection was conducted from July to September in 2013.

A total of 58 interviews were analysed, including ten semi-structured interviews (SSIs) with formal CTC providers,^1^ 16 SSIs with informal CTC providers,^2^ 24 in-depth interviews (IDIs) with MR clients, and eight key informant interviews (KIIs) with doctors, paramedics, and programme staff of the NGO clinics. MR clients were purposively selected based on the number of children they had (women who had never given birth- *n* = 4, women who had had up to two children- *n* = 10, and women who had had three or more children- *n* = 10) and educational status (*n* = 6 from each of the following educational categories: no formal schooling, 1 to 5 years of formal schooling, 6 to 10 years of formal schooling, and more than 10 years of formal schooling.). Our aim was to select an equal number of women from each of these categories, but because of the sensitivity of the issue, it was difficult to recruit women who had never given birth. CTC providers were purposively selected from a list generated through a mapping of CTC SRH service providers based on their popularity among the women in providing SRH services in the study sites. Data was collected by a team of 13 trained data collectors, all of whom held Master’s degrees in social science and had previous experience in qualitative data collection. Detailed notes were taken during each session and were used to create complete transcripts in Bangla, which were then translated into English.

Table 1 describes the various types of formal and informal CTC providers who were interviewed in the present study, with definitions adapted from the 2007 Bangladesh Health Watch [18]. Table 2 provides a detailed summary of all interview types and respondents.

**Table 1:** Training and type of services provided by interviewed formal and informal CTC healthcare providers in Bangladesh

Provider	Training	Type of services provided	Health Sector
Informal providers			
Traditional healer (*Kabiraj*)	Mostly self-trained, but some may have training from government or private colleges of ayurvedic medicine	Ayurvedic, based on diet, herbs and exercise etc. Sometimes also combine allopathic medicine such as antibiotics and steroids etc.	Private/Public
Drug store salespeople/Drug vendor/drug seller; also village ‘quack’	No formal training in dispensing; none of them are trained in diagnosis and treatment; some learn treatment through apprenticeship or working in drug stores (‘quack’)	Allopathic; in addition to dispensing, they also diagnose and treat	Private
Village Doctors (Rural Medical Practitioners, RMPs, and *Palli Chikitsoks, PCs*)	Majority (RMPs) have three to six months training from semi-formal, unregulated private organizations. Few (*PCs*) have had one year training from a short-lived government programme in the early ‘80s (*PC* training programme) which stopped in 1982.	Allopathic	Private
Traditional Birth Attendant (*Dai*)	No training or short training on safe and clean delivery by government, private organizations or NGOs	Assisting normal delivery	Private
Formal providers			
Family Welfare Visitor (FWV)	1 ½ years training in government/private facilities on midwifery and clinical contraception management	Conducting normal delivery; clinical contraception and immunization services	Public/private
Community Health Workers	Training on basic curative care for common illnesses and preventive health by government/private organizations or NGO of varying duration	Allopathic: curative and preventive/health promotion	Public/private/NGOs

**Table 2:** Types of Interviews and Respondents

Interview Type	Number of Interviews	Types of Respondents (per site)
In-Depth Interview (IDI)	6 per site × 4 sites =24 total	*Conducted among those who went through an MR experience in the past one year:* - 1 with women who have never given birth - 2/3 with women with one or two children - 2/3 with women with 3 or more children
Key Informant Interview (KII)	2 per site × 4 sites = 8 total	- 1 with medical personnel directly involved in MR (doctor/paramedic/nurse/counsellor) - 1 with programme staff (e.g. programme manager)
Semi-Structured Interview (SSI)	*Formal CTC Providers*: 2 per Dhaka site 3 per Sylhet site = 10 total	At least 1 with Government health worker At least 1 with NGO health worker
	*Informal Providers*: 4 per site × 4 sites = 16 total	*Respondents drawn from 4 categories*: *-* Traditional healer (*Kabiraj*) - Traditional birth attendant (*Dai)* - Village Doctor - Drug Seller

### Analysis

English transcripts were manually coded using the software programme Atlas.ti 7.0, with a focus on the portions of the transcripts relating to mobile phone technology. Standard qualitative research practices were used to identify themes pertaining to awareness, usage and perception, and advantages and disadvantages of mobile health services [19].

## Results

### MR clients

#### Demographics

All 24 MR clients interviewed were married women. They ranged from 17 to 42 years in age, with an average and median age of 29 years. Twenty-three of the 24 women were housewives and practiced Islam, while the last was Hindu and was employed in the service industry at a college. Four women did not have children, while the remaining 20 respondents had an average of three children. The education levels ranged from being uneducated (five women) to holding a master’s degree (one woman). Nearly half of the women (11 respondents) had not been educated past primary school level.

#### Client awareness

Nine of the 24 MR clients interviewed stated that they were aware of how mobile phones could be used to obtain health care services and information. Of these women, only three reported having used their mobile phones to consult and seek advice related to MR services. Five MR clients expressed concerns about the lack of publicity for mobile health services for women, and only two reported having seen government and private organisation campaign advertisements in visual media for either general mobile health services or for mobile health services pertaining specifically to MR. The remaining clients were unaware of formal or informal mobile health services for MR. Many of these women stated that they learned about menstrual regulation from a family member or friend, rather than from a health care provider or through a mobile health service. One of these women recognized the value of mobile phone services for MR clients, but stated that public awareness is low:

I learned about MR from my sister [referring to a close female friend]. I would not have known if she did not talk about it. She told me because she is like my own sister. This thing you said about the mobile phone service, they [service providers] should let people know about it. Then it will be easier for [women] to get [MR] services… How can [women] call if they do not know about it? They [women] need to know the phone number [to call for MR service information]. How can they call if they do not know the number?-(Sylhet, Urban site, IDI Client 2: 35 years old, married house wife, five children, Islamic, completed primary education)

In reference to mobile health services for MR, another client expressed a positive outlook despite her lack of awareness of the availability of such services:

It will be great. I will be able to understand [about MR]. If I get to know [about mobile health services] I will inform others too. - (Dhaka, urban site, IDI Client 6: 30 years old, Married house wife, three children, Islamic, basic reading and writing)

The statements above reflect the general lack of awareness observed among MR clients in all four study sites regarding the mobile health service system, indicating that there is inadequate dissemination of information about formal and informal mobile health services from both the government and local or private health organisations. As Client 2 states, greater efforts need to be made to disseminate mobile health service contact numbers to women so that they can utilize these services at times when menstrual regulation procedures may be warranted. Additionally, as Client 6 indicates, community members may also be able to help spread information on mobile health services through word-of-mouth. There is a greater need to inform the mass population, and options such as hotline contact numbers could be a means of providing general and MR health service information to women. Given the stigma associated with MR, using formal mHealth services or using mobile phones informally to seek health care can help ensure women’s privacy regarding obtaining MR service information.

#### Client usage and perceptions of mHealth

Seven of the 24 women interviewed stated that they have used their mobile phones in a health context. Reported types of usages included obtaining prescriptions, obtaining information about general diseases, and obtaining information about reproductive health. Three of the seven clients mentioned that they also used their mobile phones to obtain health information for family members, including one client who described obtaining counselling related to contraceptive services for a relative:

My sister-in-law got that 3-month injection [the Depo-Provera contraceptive shot] and became sick afterwards. Then I called Shima Apa [formal CTC provider] and let her know. She then told my sister-in-law to get ‘stick’ [IUD] inserted. Then my sister-in-law got ‘stick’ inserted. My sister-in-law is quite well now. -(Sylhet, urban low-income settlement, IDI Client 4: 23 years old, married house wife, one child, Islamic, educated through class 5)

Another client stated that she obtained counselling related to MR services using her mobile phone, which enabled her to receive a safe and timely procedure within the legal gestational window:

I called and asked for some suggestion [advice] about my problems before coming to Marie Stopes. They told me to visit the Marie Stopes clinic as early as possible because doing MR after two months is risky. - (Sylhet, rural low-income settlement, IDI Client 4: 33 years old, married house wife, three children, Islamic, Bachelor of Arts degree complete)

When asked directly about their perception of mobile health services, the majority (18 out of 24) of the respondents said that they understand the effectiveness of the services, and believe that they are essential for people who face emergency reproductive health situations. Four of these clients mentioned that using mobile phones to obtain information about safe MR is important because many women are forced to resort to informal providers for unsafe abortion if they wait past the legal gestational window for MR. The remaining six clients did not comment on this topic.

Six women stated that they were aware that they could use their mobile phones informally to obtain post-procedural care information following their MR procedure. Five of these women stated that although they were given the phone number of the NGO clinics’ health staff member who performed their MR procedure, they did not call to obtain advice because they did not experience any complications. The one woman who did use her mobile phone to obtain post-procedural advice reported both a good health outcome and general satisfaction with mobile health services:

The sister [MR provider at a clinic]… I called to tell her [about post-procedural pain]. She told me to apply hot water compression and to come for check-up if I have time. So, I have come today, to check if everything has been cleared correctly. Today I am relieved, now there is no problem. (Sylhet, rural low-income settlement, IDI Client 1: 31 years old, married service holder, one child, Hindu, Master’s degree complete).

#### Perceived advantages and disadvantages of mHealth services: clients

When asked about the general benefits of obtaining health service information through mobile phones, all 24 MR clients expressed an overall positive impression regardless of whether they knew about or had used mobile health services in the past. The perceived benefits can be broken down into the following three categories (A-C):

A. Financial Costs: Five out of 24 MR clients stated that obtaining health-related advice over the phone saved them the price of a medical visit and enabled them to avoid paying the cost of transportation to a clinic. Two characteristic responses were:

It will be a very trouble-free option for women in the future. There is no transport cost. No expense at all. (Sylhet, urban low-income settlement, IDI Client 2: 35 years old, married house wife, 5 children, Islamic, primary education complete)

I will not have to go to the doctor. I will not have to spend rickshaw [transportation] fare! - (Sylhet, urban low-income settlement, IDI Client 6: 28 years old, married house wife, 2 children, Islamic, uneducated)

B. Timeliness: Nine out of 24 MR clients felt that mobile health services could enable them to receive services more rapidly, could save them the time needed to travel to a far-away clinic (in cases where a visit was not necessary), and could reduce the waiting time at a facility by enabling them to make an appointment in advance. Two characteristic responses elucidating these sentiments were:

At times it is not possible to come to the clinic [due to] the shortage of time, [especially] if I am living far away [and] I need to get service quickly… Then I can consult [with a healthcare provider] over the phone. (Sylhet, urban low-income settlement, IDI Client 1: 31 years old, married service holder, 1 child, Hindu, Master’s degree complete)

It [mobile health services] is a good approach and it will be better [because] we can come [to the clinic] after communicating [with a healthcare provider] earlier, then no time would be required for waiting.” - (Dhaka, urban low-income settlement, IDI Client 2: 24 years old, married house wife, 2 children, Islamic, Secondary School Certificate incomplete)

C. Accessibility: Thirteen out of 24 clients emphasised that mobile health services would increase access to medical information for women who live far from a clinic and may have limited access to information or services. Additionally, they stated that mobile health services could increase access for women who cannot easily leave their homes due to household duties or to a general lack of independence. Two clients stated that mobile health services specifically for MR would be helpful for women of all classes, regardless of socioeconomic status:

If you can provide [MR treatment advice] through mobile [phones] it will be good. There are many women who are sick, who can’t visit the doctor, who don’t know what to do. Then if they could [obtain treatment advice] over mobile phone that will be good; they will not face any problem. - (Dhaka, urban low-income settlement, IDI Client 1: 17 years old, married house wife, no children, Islamic, Higher Secondary School Certificate complete)

However, five clients who attempted to informally use their mobile phones to obtain information about MR expressed frustrations with the service. Despite calling the contact number that they were given by their health care provider, these women were unable to reach a provider or counsellor over the phone and thus could not utilize the mobile health service.

As I couldn’t reach them [healthcare providers] over the phone, I had to come here [the clinic]. I came here for an MR procedure. They checked me up well and asked to do MR and prescribed medicine. Later on when I felt pain in my navel I called on mobile phone, but no one [picked up]. –(Sylhet, rural low-income settlement, IDI Client 6: 28 years old, married house wife, 2 children, Islamic, uneducated)

#### Summary

As a whole, our study sample of Bangladeshi MR clients from low-income settlements expressed an overall positive perception of MR-related mobile health services, with numerous perceived benefits. While the majority of the clients we interviewed believed that mHealth services would be effective and beneficial with respect to disseminating information on MR, many had never used these services before and some chose not to comment on the topic. Most clients agreed that mHealth services for MR would be beneficial to women with respect to financial costs, timeliness and accessibility of services. These women voiced that regardless of demographic backgrounds, mHealth services could enable women to more easily access doctors or health professionals for proper counselling in times of health crisis. However, some women raised concerns that information dissemination about both formal and informal mHealth services needs to improve, as many women are unaware of the ways that they can use their mobile phones to obtain health-related information or counselling.

### CTC providers

#### Formal providers: types of mobile health services provided

Ten out of the 18 formal providers interviewed gave similar responses about the types of services offered by their clinic over the phone. These services included general disease inquiry, counselling on SRH issues such as menstruation, sexual complications, vaginal infection, and menstrual regulation, verbal diagnosis of sexual diseases, nutrition profiles for pregnant mothers, and making appointments.

#### Formal providers: perceptions of mHealth

The formal providers had varied opinions about mHealth services. Thirteen providers stated that mHealth services can be useful for counselling clients, particularly MR cases. However, four of the providers stated that speaking with clients over the phone is insufficient for counselling on MR, and that often times it is necessary to physically examine a client before making medical recommendations:

There are differences between physical check-ups and mobile phone conversations. There is a huge gap between these. For example, if [a client] says that she is having a white discharge then we will not understand the reason just by [speaking to] her over the phone. But [the cause] can be identified through physical check-ups… [to] confirm whether or not she has any sexual diseases. –(Health care programme manager, Dhaka, KII respondent)

Many providers mentioned clinic “help lines” that are available for clients to call to obtain information on SRH services, including MR. While 10 formal providers found the helpline service system to be effective because it enables clients to receive information and advice without visiting the clinic, they expressed frustration with the fact that at times they have to receive calls and consult with clients at inconvenient hours. One such provider stated:

I just give advice over phone. Clients ask over phone about different problems, what to do, where to go, etc. I tell them to take care of themselves. Again… if after doing MR they face any problem, then I give them advice over the phone for some common problems. I suggest the pregnant mothers to take proper diet, to take nutritious food, to take enough rest. I tell them to come regularly for check-up. Sometimes I also call them to ask about their health condition. –(CTC formal provider, Dhaka, SSI respondent: Family Welfare Visitor, female, 43 years old, 23 years of practice, higher secondary education, Islamic)

#### Informal providers: perceptions of mHealth

Like the formal CTC providers, the informal providers interviewed also expressed hesitations about the extent to which health services can be provided over the phone. Of the 16 interviews performed within formal providers, four *Kabiraj,* five *Dai* and two drug store salesmen stated that it is not possible to provide all types of treatment over the phone. However, many did express that mobile phones can be useful for coordinating with clients:

[Healthcare provision] does not work by using phones. Clients must come here. But if someone calls me then I tell them a time to come and meet me.–(CTC informal provider, Dhaka, urban low-income settlement, SSI respondent: *Dai*, female, 65 years old, 50 years of practice, uneducated, Islamic)

One *Dai* stated that she provides various services over the phone, most of which are consultations:

I only give service in two places over mobile phone; one in my own house, and my in-laws house. If any client now lives far away or is older then I give service over phone. If any previous client calls, then I ask for a few details and then give [medical advice]. There are many who get advice over phone. – (CTC informal provider, Dhaka, urban low-income settlement, SSI respondent)

#### Perceived benefits and disadvantages of mHealth services: all providers

Considering the responses of both formal and informal CTC service providers, we categorised the perceived benefits and disadvantages of mHealth services as following (A-C):

A. Financial Costs: Both formal and informal providers stated that they have to bear the cost of their mobile phone credits in order to communicate with their clients. All expressed that providing a mobile phone service makes their clients feel content and enables both current and new clients to contact them regarding community health services. However, all formal providers, including governmental ones, mentioned that mobile phone costs are not usually included in their salary. All providers said that they do not mind paying the phone bills as long as it is for a good cause that is serving the community, as exemplified by this formal provider’s statements:

I spend my own money for phone calls. Office does not [reimburse me for] that expenditure. Even if any client calls me then I cut the line and call back from my phone. If I do so, client will say I am kind to them. If I spend ten Taka [Bangladeshi currency] in this way, then I will be able to earn twenty Taka in future. If she is cured with my treatment or with my advice, then she would send more clients to me.–(CTC formal provider, Dhaka, urban low-income settlement, SSI respondent: NGO Community Health Worker, female, 30 years old, 9 years of practice, Islamic, primary education incomplete)

B. Timeliness: The formal providers we interviewed believed that mHealth services can be effective for time management. Ten of the formal providers said that speaking with clients on the phone saves time in clinic and enables them to give pertinent health information to clients in a timely manner. Informal providers also agreed that in times of crisis, mobile phone services are beneficial for giving valuable information to clients. The majority of the informal providers understood that proper timely treatment can avoid fatalities in MR cases. Six of the drug store salesmen mentioned that clients call them at times with questions about MR complications and which medicines they should take to treat them. The following responses illustrate these sentiments:

[Mobile health services] would be better in terms of time. If a problem happens to a client, then I could have a conversation with the doctor or compounder, could give advice. If necessary, the client could be brought to hospital through a phone call. – (CTC informal provider, Sylhet, rural low-income settlement, SSI respondent: *Dai*, female, 79 years old, 10 years of practice, Hindu, uneducated)

I can consult with them quickly. I can provide the service in less time.–(CTC formal provider, Sylhet, urban low-income settlement, Family Welfare Visitor, female, 40 years old, 21 years of practice, Hindu, higher secondary education complete)

C. Accessibility: Eight formal providers agreed that use of mobile health services is effective in terms of increasing clients’ access to general health information:

[Mobile health service] is very effective. [Clients] get quick service and it is accessible. When it is not possible to come [to the clinic] then women can get [counselling] through mobile phone. Suppose a woman is having heavy bleeding; then she can call and ask for advice. We can suggest her to take this medicine or take rice saline. We can also get information [about] the current status of the client. In severe cases we can refer the client to the hospital or any specific place.–(CTC formal provider, Dhaka, urban low-income settlement, SSI Respondent: Family Welfare Visitor, female, 42 years old, 25 years of practice, Islamic, Secondary School Certificate complete)

However, four informal providers also believed that mobile health services alone are not sufficient for giving advice regarding MR. These providers emphasized that it is important to understand each client’s medical condition through in-person consultations:

Sometimes [counselling] can be taken through phone. But there are problems [with doing so]. Will it be right to give treatment without seeing the client? It might happen that I can’t understand what they are saying in the phone. Then instead of helping the client I might harm her. – (CTC informal provider, Sylhet, rural low-income settlement, SSI respondent: *Dai*, female, 79 years old, 10 years of practice, Hindu, uneducated)

#### Summary

Overall, the formal and informal providers of MR services that we interviewed perceived mHealth services to be effective in terms of information dissemination with respect to SRH generally and MR specifically. Formal providers also use it for referring clients. However, some providers felt that mobile health services cannot meet all health needs, particularly in the event of serious clinical complications that require an in-person consultation or hospital visit. In particular, the informal providers that we interviewed only wanted to use mobile health services to inform clients of their availability and how they can be reached in times of health crises. Although both formal and informal providers bear the financial costs of communicating through mobile technology without any extra monetary compensation from their respective organisations, providers generally felt that doing so enhanced their service delivery and viewed it as a service to the community.

## Discussion

Despite the expansion in mobile health care services worldwide [20] and in Bangladesh specifically [21], our findings suggest that low-income clients reliant on close-to-community care providers in Bangladesh have an inadequate understanding of how to use their mobile phones to obtain health service information or counselling, especially pertaining to MR. The majority of the MR clients we interviewed were unaware of the existence of the various formal mHealth service systems in Bangladesh, and had no knowledge of the “helpline” systems which formal CTC providers claimed to be in place in the study areas. They also did not generally use their mobile phones informally to obtain SRHR service information or counselling related to MR. These findings corroborate those of a similar study conducted in Chakaria sub-district of Bangladesh [14], where only 1% of community members knew about “helpline” service options. Likewise, a study conducted in Bangladesh found that while the government service providers generally are aware of the mHealth service delivery system, service recipients are not well-informed and hence have a poor perception of it. Possible reasons for low awareness could include the poor functional literacy rate in Bangladesh (45.3%), and the fact that the majority of the population lives in the rural areas and hence relies mostly on the traditional or low-tech options to access health related information [21].

Among MR clients who did report using their mobile phones for health-related purposes in the past, the most common motive was to obtain general health information or make appointments, with only a few contacting service providers specifically for MR-related counselling. This finding is in line with the results of a previous study conducted in another urban slum in Dhaka, which found that community members used their mobile phones to call and secure appointments with doctors but did not generally use them to seek medical advice or information unless they had personal connections with service providers [22]. Additionally, the participants in that study generally first contacted relatives and friends for health information before calling service providers, a tendency that was also common among the participants in our study sample with respect to sexual health information.

Despite the low levels of awareness and usage of mobile health services among the MR clients in our study sample, the majority expressed a belief that mHealth services targeted towards MR would be effective and helpful for women who have limited access to health care clinics, be it due to geographical distance or due to a lack of the independence necessary to travel to a clinic. All of the formal and informal MR providers that we interviewed also agreed that mHealth can be an effective means for delivering health information, providing proper counselling, and counselling clients in times of health crises. Our findings furthermore suggest that formal providers of MR services consider mHealth to be particularly useful for clients who live far from health care centres, as it saves time and is cost-effective. Both informal and formal providers reported having to bear the financial costs associated with mobile health services personally, with no compensation or subsidies either by the government, NGO or from the private organisations. However, none of the providers we interviewed had any complaints about this financial aspect, as they perceived their mobile service provision to be for a good cause.

Formal and informal mHealth services present a promising opportunity for increasing health-seeking behaviour among women living in resource-poor settings in Bangladesh, and may be an effective means of increasing access to safe, formal menstrual regulation services among women who might otherwise resort to unsafe abortion. However, challenges remain with respect to scaling up access to mHealth services among marginalised women in Bangladesh. The results of the present study highlight the fact that there is a need for greater publicity of both formal and informal mHealth services targeted towards SRHR in slum communities in Bangladesh, particularly with respect to hotline systems and clinic numbers that clients can call to seek information about formal, safe MR services. Furthermore, there are additional barriers to ensuring that women have access to the mobile phones needed to utilize mHealth services. Globally, women are 21% less likely to own a mobile phone than men, and this gender disadvantage is the highest in South Asia [23]. Additionally, the ability of married women to use mobile phones in order to seek health information is often restricted by their husbands’ control over their phones and finances [24].

## Conclusions

Our findings have implications for the design and improvement of mHealth intervention frameworks in Bangladesh and other low-income countries, particularly with respect to stigmatized health services such as menstrual regulation and abortion. We report a low level of awareness and utilization of mHealth services among MR clients in slum communities in Bangladesh, but widespread optimism about the potential for mHealth to increase timely access to formal providers of MR among abortion-seeking women. Our findings also reaffirm that healthcare providers still perceive face-to-face interaction as crucial and important for proper diagnosis and treatment of SRHR health issues, especially MR. Thus, while expanding formal and informal mHealth services for SRHR in Bangladesh may be useful in disseminating information about MR and connecting women with formal providers, in-person visits remain necessary for adequate treatment. Further studies could investigate cost-effective ways to implement and publicize formal and informal mHealth services for MR and other stigmatized SRHR topics among reproductive-aged women living in low-income settlements, as well as the impact of these services on unsafe abortion rates. Policy makers may use the findings from this study to improve on the accessibility and user acceptability of mHealth services both by health staff and health consumers.

## Abbreviations

CTC: Close-to-community
IDI: In-depth interview
KII: Key informant interview
mHealth: Mobile health
MMR: Maternal mortality ratio
MR: Menstrual regulation
NGO: Non-profit organization
PC: *palli Chikitsok*
RMP: Rural medical practitioner
SRH: Sexual and reproductive health
SRHR: Sexual and reproductive health and rights
SSI: Semi-structured interview

## Footnotes

1 Providers who are affiliated to any kind of institution and have recognized qualifications from any formal, registered and regulated government, NGO or private institution are considered formal CTC providers [18].

2 Health service providers who are not affiliated to any formal institution or registered with or regulated by any government regulatory body are considered informal providers [25].
